# Cooperation of the Public Schools in Teaching "Good Teeth, Good Health."

**Published:** 1904-08

**Authors:** Richard Grady

**Affiliations:** Dentist U. S. Naval Academy.


### COOPERATION OF THE PUBLIC SCHOOLS IN TEACHING "GOOD TEETH, GOOD HEALTH."

By Richard Grady, M. D., D. D. S.

Dentist U. S. Naval Academy.

(Read before the American Medical Association, Atlantic City, N. J.)

"Whatever we wish to see introduced into the life of a nation must first be introduced into its schools."

In my office there is a motto, "Good Teeth, Good Health," suggestive of the central thought of this paper. In many cases it has made a deep impression upon the mind of the patient whose eye has caught the inscription as he entered the room, and he has been prompted to read the lines attached to it:

Without good teeth there cannot be thorough *mastication*.

Without thorough mastication there cannot be perfect *digestion*.

Without perfect digestion there cannot be proper *assimilation*.

Without proper assimilation there cannot be *nutrition*.

Without nutrition there cannot be *health*.

Without health what is *life?*

Hence the paramount importance of the teeth.

It is a noteworthy fact that a large proportion of my patients are boys and girls from six years of age upward, and young men from fifteen to twenty-four years of age. For the past twenty years I have been visiting dentist to schools and public institutions in addition to caring for children in my office practice.

The examination of many mouths has given me an opportunity such as few, if any, dentists have had of noting the condition of the teeth of young people, so that I ought to have a speaking acquaintance with my subject. It would be difficult, in my opinion, to find a like number of children in any public school (and I speak from a varied experience of nineteen years as a teacher) in as good physical condition as those who have received systematic care of the teeth. A few years ago I discovered that not one of the 500 children in one of the public institutions in my city had a toothbrush. The children of that institution are now supplied with toothbrushes, but I fear there are many similar institutions in the world where the toothbrush is never seen and the dentist never heard of.

There is a growing demand that the subject of hygiene should receive increasing attention in general education. Happily it requires less knowledge to keep what we already have than to recover it when lost. How to take care of the health or to avoid many causes of diseases may be learned. It has been estimated that half of the children born into the world die before reaching the age of sixteen. We can truthfully charge a large share of this great slaughter to ignorance or wilful neglect.

The efficiency of prevention is proverbial. Pain is undoubtedly one of the most dreaded accompaniments of physical ills. I am fully persuaded that more than fifty per cent. of dental caries is absolutely preventable by medicines internally administered which act specifically in the mouth; alike pleasing and imperative is the appeal to both ambition and intelligence to prevent, rather than to repair, the ravages of decay.

Is it right, therefore, that the vast research respecting the hygiene of the mouth and control of dental disease, which has employed practitioners of dentistry for years, should go for nothing in education; that this wealth of knowledge should be passed by as if it had no existence, and the young people of the country grow up as ignorant of it as if they had lived centuries ago? The problem which the science of dentistry is endeavoring to unfold, and the end which it aims to attain is to prevent the bacterial destruction of tooth-structure. If the teeth are not allowed to accumulate deposits on either their exposed or protected surfaces, they will, it is claimed, be exempt from caries. More broadly stated the propositon is: Given the varying predisposition of different individuals to caries, which is governed by the laws of heredity and environment, the growth of micro-organisms in the mouth is in proportion to the amount of disturbance they suffer, or rest and opportunity they enjoy. This is a recognized fact and almost axiomatic.

A large amount of suffering may be avoided through proper knowledge conscientiously applied. It would be a great saving of "Young America," and thereby of all America, if every board of health imitated the example of Ontario's board, which adopted the following resolution: "That dental inspectors be appointed by local boards of school trustees to periodically visit schools and examine children's teeth, and that a dental hospital be started in Toronto for the benefit of poor children; and these recommendations be urged upon the attention of the Minister of Education."

It will thus be seen that school boards have been urged to take the matter up. But treating diseased teeth of school children at public expense seems entirely out of the question at present; yet why should it be less reasonable to have visiting dentists than visiting music teachers, and drawing teachers, and teachers of physical culture? The position of visiting dentist in our public schools would not be a sinecure. There would be work to do every day. Just how far he should be armed with authority might be variously decided by different school boards. In general, it may be premised that his care and attention to any individual pupil should end where such pupil's physical condition ceased to be a threat or an offense to the health of his fellows. The State, however, at once can touch the subject by attending to the question of prevention. If bad teeth could be prevented the gain to the State and the individual would be of enormous value, as it is wonderful how many diseases can be traced indirectly to bad teeth.

It is the noble privilege of the teachers of the country (who are acknowledged as safe guides and who aspire to possess an amount of physiological knowledge such as every educated person ought to possess) to promote in some degree the preservation of the teeth of those under their care; and this they can do by inculcating early and earnestly and with the emphasis of a high religious duty the principles of oral hygiene. It is a subject in which all mankind has an interest, even though it be, as it too often is, an unconscious interest. The life of every man, woman and child ought to be guided and governed by its laws. This being so, the subject should be presented and agitated in many forms until its importance is appreciated. And not the least of the benefits which will follow the better diffusion of physiological and sanitary information will be the protection of the community from the impostures of charlatans and also discrimination in the qualifications of dentists.

Side by side with medical science, dentistry has grown. Now the teeth are recognized as important objects of medical study as the eye or ear. The same spirit which has led to enlisting the active cooperation of the public schools concerning measures for preventing the spread of contagious diseases of childhood should be insisted upon in the care of the teeth. A movement of physicians to secure public action on this matter will at once exert a moral force. Mothers will be more watchful and painstaking to avoid the disgrace of decayed teeth in their children's mouths. Children will be moved to take a pride in spotless teeth; and in this age to acquire an ambition to have anything white and spotless about the person is commendable.

School life and conditions regarding caries are at their worst in our great cities and require the most urgent amendment. The inculcation of cleanly oral habits among children should be insisted upon. The hope of dentistry is to employ prophylactics instead of remedies to prevent decay instead of treating it. It is just as important and as necessary to keep the teeth clean as it is to keep the hands and face clean, because all the food we eat must come in contact with our teeth. We do not like to eat when our hands are unclean, and why should we take pleasure in satisfying our appetites when our teeth are unclean. It is essential that a child be taught how to brush the teeth properly, and for this purpose I keep in my cabinet a toothbrush and set of teeth mounted with which I give a careful object-lesson. Printed directions are attached to the brush: "Brush the upper teeth downward and the lower teeth upward on their inner and outer surfaces, preventing injury to the gums, and effectively cleaning all the crevices of the teeth." A toothbrush drill at school is as needful as any gymnastic exercise for the preservation of health. There is strong reason to believe that many diseases of the nervous system, respiratory organs, and alimentary canal, may be due to the fact that the masticatory organs have been neglected.

A paper on the theme of which I write would a few years ago have excited but little interest. To-day parents, teachers, influential citizens and even professional men, are being educated to the full comprehension of the fact that of all diseases of a parasitic nature to which mankind is susceptible, dental caries is by far the most frequent. The only proper course to pursue in dealing with this question is by persistently and intelligently educating the minds of the public as to its exact status, and whatever we wish to see introduced into the life of a nation, we must remember, must first be introduced into its schools. The school is the one force that can unify all conditions of society. Here we have the children of the nation in their entirety and we can if we will teach them that soundness of teeth is in itself one of the best evidences of general soundness of body.

We are now able to say to teachers and pupils that it has been proven beyond doubt that decay of the teeth is progressive—first stage chemical, second, parasitical; that the prevention of dental caries depends, first of all, on strict cleanliness of the mouth and teeth, the importance of which cannot possibly be overestimated. The details necessary for the proper fulfilment of the same must be given in textbooks, in public talks and in the public press. Undoubtedly, it can be said that the toothbrush and plenty of clean water stand at the head of all measures of this nature, and that the next prophylactic means is the intelligent use of proper antiseptics. A tepid salt solution has been recommended as an inexpensive and effective antiseptic for rinsing the mouth, and where there is a tendency to bleeding of the gums powdered boric acid may be used. One thing not often spoken of in reference to cleaning teeth is the value of rinsing. Many patients know nothing about it and the average dentist does not think it worth while to mention it to them. The matter of closing the lips and forcing the water vigorously back and forth between the teeth exercises an important part in cleansing them.

An elementary outline of dental science, in language plain and intelligible, embodying a brief exposition of the actual present state of knowledge accepted as authoritative, should be taught to, and studied by, all pupils of the public schools whose capacity will admit of it, and in the same manner as other like branches are taught and studied, if necessary with text-books in the hands of the pupils.

We want to emphasize the startling fact that the teeth of children have been deteriorating to such an extent that it has now become a serious matter. Of all causes of decay uncleanness is perhaps the most fertile. The clean tooth may decay—the neglected tooth must decay. It has lost its chance of self--defense.

Irregularities of the teeth, as shown by Dr. E. S. Talbot, are also on the increase, and there seems to be no plan by which the increase can be checked unless it be in the care of children's teeth and the observance of common-sense rules regarding extraction of the adult teeth. Check in the beginning irregularities which might prove mischievous if left unattended.

We want children instructed in the care of the mouth and teeth, in cleansing the whole mouth including the tongue, and the sooner this is done the sooner will the many evils arising from the present neglect be stayed. Children must be taught some system of oral hygiene. See that school children receive thorough instruction as to the utility of good teeth. The teaching which a growing child imbibes in school sticks fastest in its memory. "My teacher told me to do it that way." Young minds are very susceptible and they would readily understand the situation, especially if told the calamities liable to follow the neglect of their teeth and mouths. These same children will soon be the parents of the community in their turn, and they would have the advantage not only of better mouths of their own, but of being in a position to care for their children. What has been done in the treatment of zymotic diseases, namely, an improvement in the condition of surroundings, is precisely what is required respecting the teeth. Instead of regarding the teeth as foes, children should be taught to regard them as special friends, and devote to them their best care.

We want to remove the possibilities of the propagation of disease in public schools through the present condition of children's mouths and teeth, and incidentally the discomfort of parents whose children have aching teeth, sleepless nights, distorted nervous systems, bad digestion, alveolar abscesses, foul and fetid breaths. When children are in this oral condition the whole system is out of order and they are more susceptible to disease.

Answering the question, why do we want instruction and inspection to prevent sacrifice of children's teeth, and the accompanying effects on their health, I would say:

1. Take it all in all, care of the teeth pays in comfort, in beauty, in the conservation of health from youth to old age. "Better take pains than to have pains take you."

2. It has been demonstrated that 95 per cent. of children have their permanent teeth decayed, ranging in number from two to sixteen per child. The magnitude of the evil appears at the simple mention of the fact. Can children with carious teeth grow into healthy adults? Can a race thrive whose children are so afflicted? When one has attained full growth it may not matter much whether the food is masticated by natural or artificial means, provided it is properly done; but with children it is a different matter, and the state of our children's teeth is a question of national importance. In chronic indigestion only two persons in one hundred have sound teeth.

3. Relatively, few children have teeth filled, and those under ten years of age rarely have the dentist's care except for the extraction of loose and aching teeth. Why is it that so large a percentage of our children, school-children, present features distorted, often due only to untimely, injudicious loss of teeth, deciduous and permanent? Because of ignorance or neglect.

4. The teeth and mouths of many children are in an unhealthy and disgusting condition, which not only injures their own health but also the health of the teachers and other children who are compelled to sit with them, it may be in overcrowded or ill-ventilated rooms. It ought not to be difficult to impress teachers with the dangers which attend the exuding of pus from abscessed teeth. In every community there are those who are enthusiasts on the subject of pure and wholesome food, but whose mouths are in such a neglected condition that the air which passes through them is polluted and every mouthful of food swallowed carries bacteria with it into the stomach. The almost entire futility of sterilizing articles of diet for patients in whose mouths chronic abscesses exist, or whose teeth are covered with tartar mixed with mucus and food in a state of decomposition, need hardly be mentioned. A source of danger from decayed teeth is the possible introduction of parasites into the tissues with which the teeth are connected. Parasitic organisms are numerous in articles of food, both as usual and occasional associates, and as it is very difficult to prevent small particles of food from lodging in the cavities of carious teeth and there undergoing decomposition, it is not impossible that by such means, especially if the cavity is the root-canal of a dead tooth, a parasite might enter the soft tissues.

5. There is also another source of danger to children who exchange pencils and chewing-gum, which after being in mouths mixed with pus are placed in the mouths of other innocent and unsuspecting children. These practices may be democratic, but they are vicious.

6. Girls whose teeth are defective, in a few years will be the mothers of the next generation. What about the claims of their children unless we now do our duty by the future mothers, and give them a chance to grow up as healthy women? Dr. D. Hayes Agnew said: "The world is becoming filled with a class of flat-breasted, spindle-limbed young women, unfitted for the varied and responsible functions of womanhood; qualifications, too, which under a different regimen and directed into proper channels would exert a most potential influence on all the great social and moral problems of the age."

